# Decreases in TGF-β1 and PDGF levels are associated with echocardiographic changes during adjuvant radiotherapy for breast cancer

**DOI:** 10.1186/s13014-018-1150-7

**Published:** 2018-10-19

**Authors:** Hanna Aula, Tanja Skyttä, Suvi Tuohinen, Tiina Luukkaala, Mari Hämäläinen, Vesa Virtanen, Pekka Raatikainen, Eeva Moilanen, Pirkko-Liisa Kellokumpu-Lehtinen

**Affiliations:** 1 Faculty of Medicine and Life Sciences, University of Tampere, PO Box 100, 33014 Tampere, Finland; 2 Department of Oncology, Tampere University Hospital, PO Box 2000, 33521 Tampere, Finland; 3 Heart Hospital, Tampere University Hospital, PO Box 2000, 33521 Tampere, Finland; 4 Department of Cardiology, Heart and Lung Center, Helsinki University Hospital, PO Box 340, Tampere, 00029 Finland; 5 Research, Development and Innovation Center, Pirkanmaa Hospital District, PO Box 2000, 33521 Tampere, Finland; 6 Health Sciences, Faculty of Social Sciences, University of Tampere, PO Box 100, 33014 Tampere, Finland; 7 The Immunopharmacology Research Group, Faculty of Medicine and Life Sciences, University of Tampere and Tampere University Hospital, PO Box 100, 33014 Tampere, Finland

**Keywords:** Cardiotoxicity, Breast cancer, Radiotherapy, Transforming growth factor beta-1, Platelet-derived growth factor, Echocardiography

## Abstract

**Background:**

Radiation-induced heart disease is mainly caused by activation of the fibrotic process. Transforming growth factor-beta 1 (TGF-β1) and platelet-derived growth factor (PDGF) are pro-fibrotic mediators. The aim of our study was to evaluate the behavior of TGF-β1 and PDGF during adjuvant radiotherapy (RT) for breast cancer and the association of these cytokines with echocardiographic changes.

**Methods:**

Our study included 73 women with early-stage breast cancer or ductal carcinoma in situ (DCIS) receiving post-operative RT but not chemotherapy. TGF-β1 and PDGF levels in serum samples taken before and on the last day of RT were measured by an enzyme-linked immunosorbent assay. Echocardiography was also performed at same time points. Patients were grouped according to a ≥ 15% worsening in tricuspid annular plane systolic excursion (TAPSE) and pericardium calibrated integrated backscatter (cIBS).

**Results:**

In all patients, the median TGF-β1 decreased from 25.0 (IQR 21.1–30.3) ng/ml to 23.6 (IQR 19.6–26.8) ng/ml (*p* = 0.003), and the median PDGF decreased from 18.0 (IQR 13.7–22.7) ng/ml to 15.6 (IQR 12.7–19.5) ng/ml (*p* < 0.001). The baseline TGF-β1, 30.7 (IQR 26.0–35.9) ng/l vs. 23.4 (IQR 20.1–27.3) ng/l (*p* < 0.001), and PDGF, 21.5. (IQR 15.7–31.2) ng/l vs. 16.9. (IQR 13.0–21.2) ng/ml, were higher in patients with a ≥ 15% decrease in TAPSE than in patients with a < 15% decrease. In patients with a ≥ 15% decrease in TAPSE, the median TGF-β1 decreased to 24.7 (IQR 20.0–29.8) ng/ml (*p* < 0.001), and the median PDGF decreased to 16.7 (IQR 12.9–20.9) ng/ml (*p* < 0.001). The patients with a < 15% decrease had stable TGF-β1 (*p* = 0.104), but PDGF decreased to 15.1 (IQR 12.5–18.6), *p* = 0.005. The patients with a ≥ 15% increase in cIBS exhibited a decrease in TGF-β1 from 26.0 (IQR 21.7–29.7) to 22.5 (IQR 16.6.-26.7) ng/ml, *p* < 0.001, and a decrease in PDGF from 19.8 (IQR 14.6–25.9) to 15.7 (IQR 12.8–20.2) ng/ml, *p* < 0.001. In patients with a < 15% increase, TGF-β1 and PDGF did not change significantly, *p* = 0.149 and *p* = 0.053, respectively.

**Conclusion:**

We observed a decrease in TGF-β1 and PDGF levels during adjuvant RT for breast cancer. Echocardiographic changes, namely, in TAPSE and cIBS, were associated with a greater decrease in TGF-β1 and PDGF levels. Longer follow-up times will show whether these changes observed during RT translate into increased cardiovascular morbidity.

**Electronic supplementary material:**

The online version of this article (10.1186/s13014-018-1150-7) contains supplementary material, which is available to authorized users.

## Background

Late adverse effects of radiotherapy (RT), including radiation-induced heart disease, are mostly caused by fibrotic processes and take years to manifest [1]. Although the relationship between fibrosis and early inflammatory responses to microvascular damage caused by radiation is still unclear, it has been shown that pro-fibrotic mediators, including the fibroblast activating cytokines transforming growth factor-beta 1 (TGF-β1) and platelet derived growth factor (PDGF), are released by inflammatory, endothelial and epithelial cells [2]. Increased expression of TGF-β1 and PDGF in response to irradiation has been reported in animal and in vitro studies [3], but evidence describing the behavior of circulating TGF-β1 and PDGF in humans is varying [4–6].

High plasma or serum levels of TGF-β1 before RT have been associated with fibrosis of the breast [4, 5]. Regardless of whether patients received intra-operative RT or not, TGF-β1 concentrations in wound fluid were similar 24 h after surgery [7]. The relationship between TGF-β1 and RT has been most extensively studied in lung cancer patients. A meta-analysis concluded that the risk of radiation pneumonitis was increased in lung cancer patients receiving RT with a post-RT/pre-RT TGF-β1 ratio ≥ 1 [6]. TGF-β1 expression is also induced after a myocardial infarction (MI), but the exact role of TGF-β1 in MI remains elusive [3].

Increased PDGF levels are linked to the development of fibrosis, and PDGF also acts as a pro-angiogenic mediator [8]. In one study, serum PDGF levels declined after RT of non-Hodgkin lymphoma with varying target sites [9], and in another study, serum PDGF levels did not change after chemotherapy and mediastinal RT for Hodgkin’s lymphoma [10]. In animal studies, inhibition of PDGF or both TGF-β1 and PDGF during RT attenuated the development of pulmonary fibrosis [11, 12]. To our knowledge, PDGF has not been previously studied in relation to breast cancer RT.

The aim of our study was to evaluate the behavior of serum TGF-β1 and PDGF during adjuvant RT for early breast cancer and to find associations with changes in echocardiographic parameters.

## Materials and methods

### Patients

This observational, prospective, single-center study included 73 women with early stage breast cancer or ductal carcinoma in situ (DCIS). All patients received postoperative RT after breast conserving surgery (*n* = 72) or mastectomy (*n* = 1), but did not receive chemotherapy. The patient characteristics of the study population are shown in Table 1. The inclusion and exclusion criteria have been previously described [13]. The Tampere University hospital ethics committee approved the study (R10160), and informed consent was obtained from all participants.

**Table 1:** Patient characteristics (*n* = 73)

Age, Md (IQR; range)	64	(58–66; 49–79)
BMI, Md (IQR; range)	26.3	(24.2–29.9; 20–41), *n* = 69
Left-sided BC, *n* (%)	50	(68.5)
AI use, n (%)	26	(35.6)
Tamoxifen use, *n* (%)	6	(8.2)
ACE or ARB use, *n* (%)	22	(30.1)
ASA use, *n* (%)	8	(11.0)
Beta-blocker use, *n* (%)	12	(16.4)
Statin use, *n* (%)	15	(20.5)
CAD, *n* (%)	3	(4.1)
Diabetes, n (%)	6	(8.2), *n* = 69
Hypertension, *n* (%)	30	(41.1)
Hypothyroidism, *n* (%)	12	(16.4)
Smoking, *n* (%)	8	(11)

*Md* median, *IQR* interquartile range, *BMI* body mass index, *BC* breast cancer, *AI* aromatase inhibitor, *ACE* angiotensin converting enzyme inhibitor, *ARB* angiotensin II receptor blocker, *ASA* low dose acetylsalicylic acid, *CAD* coronary artery disease, *Diabetes* use of diabetes medication

### Radiotherapy

The RT protocol has been previously described in detail [14]. Patients received either 50 Gy in 2 Gy fractions or 42.56 Gy in 2.66 Gy fractions. The planning target volume (PTV) was the remaining breast with margins for patients with breast conserving surgery and the chest wall with margins for the post-mastectomy patient. Two patients had positive axillary nodes, and the PTV included axillary and supraclavicular areas.

### Serum biomarker analysis

Serum samples were drawn before RT and on the last day of RT, and they were stored at − 80 °C until analysis. TGF-β1 and PDGF-AB concentrations were determined with an enzyme-linked immunosorbent assay using the reagents from R&D Systems Europe Ltd. (Abingdon, UK). The detection limit and the inter-assay coefficient of variation were 7.8 pg/ml and 5.4% for TGF-b1 and 3.9 pg/ml and 4.6% for PDGF-AB, respectively.

### Echocardiographic examination

Echocardiographic examinations were performed by a single cardiologist (ST) before and at the end of RT. A commercially available ultrasound machine (Philips iE33 ultrasound system; Philips, Bothell, WA, USA) and a 1–5 MHz matrix-array X5–1 transducer were used to perform the examination, as previously described [13], in a standardized manner following current guidelines [15–18]. The patients were divided into two groups, one with a ≥ 15% decline and the other with a < 15% decline in tricuspid annular plane systolic excursion (TAPSE), as we earlier reported marked changes in these two parameters [13, 19]. The decline was chosen to represent an approximately 4-mm decrease in TAPSE, which can be considered a clinically meaningful change as in patients with pulmonary hypertension with every 1-mm decrease in TAPSE, risk of death was increased by 17% [20]. Also, in our earlier study the significant average reduction of TAPSE was 2.1 ± 3.2 mm and TAPSE decreased by 4 mm in 39% of patients [19]. Similarly, a ≥ 15% increase and a < 15% increase in the pericardium calibrated integrated backscatter (cIBS) were used to categorize patients into two groups. As the magnitude of a clinically meaningful change is not known for cIBS, a 15% cutoff was used to keep the change similar to the change in TAPSE.

### Statistical analysis

As the distribution of all continuous variables was skewed, medians and interquartile ranges were calculated. The Wilcoxon signed-rank test was utilized to test for changes in the biomarkers and the echocardiographic parameters from before to after RT. To test the linear relationships among the biomarkers, Spearman’s correlation was used. The patients were divided into two groups for further analysis according to a 15% change in TAPSE or cIBS as described above. To test for differences in patient characteristics, biomarker levels and radiation doses between the described groups, Fisher’s exact test for categorical variables, and the Mann–Whitney U-test for continuous variables were used. Multivariable logistic regression was used to test the change in TGF-β1 or PDGF and the change in TAPSE and cIBS using age, use of hypertension medication and mean heart dose as predictors. IBM SPSS statistics for Windows (version 23, IBM Corp., Armonk, NY, USA) was used for all statistical analysis. *P*-values less than 0.05 were considered statistically significant.

## Results

### TGF-β1 and PDGF

**Fig. 1:**
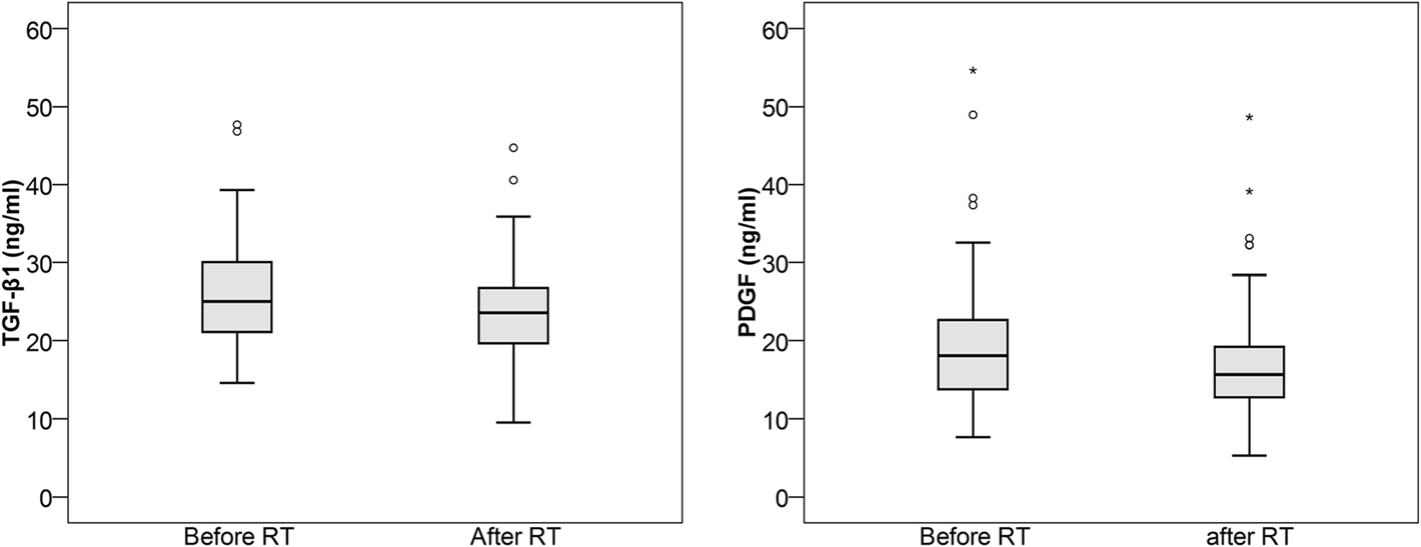
TGF-β1 and PDGF levels decreased significantly during RT, *p* = 0.003 and *p* < 0.001, respectively

**Fig. 2:**
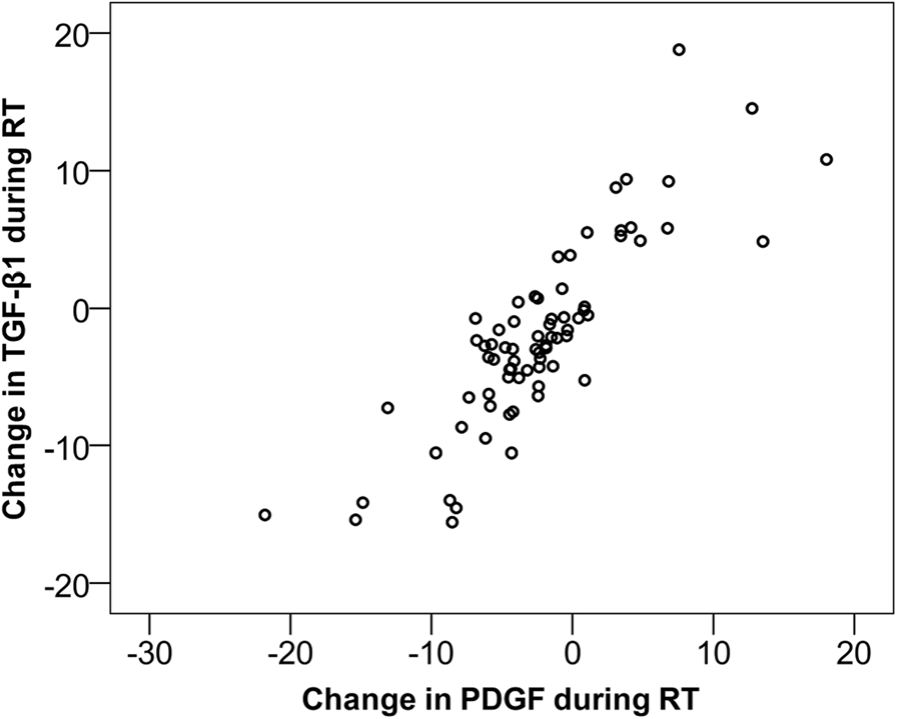
Correlation of the changes in TGF-β1 and PDGF levels during RT (Spearman’s rho = 0.817)

The TGF-β1 and PDGF levels of all 73 patients were measured before and after RT. In these patients, the median (Interquartile Range; IQR) TGF-β1 levels decreased from 25.0 (IQR 21.1–30.3) ng/ml before RT to 23.6 (IQR 19.6–26.8) ng/ml after RT, *p* = 0.003 (Fig. 1). Similarly, the median PDGF levels decreased from 18.0 (IQR 13.7–22.7) ng/ml before RT to 15.6 (IQR 12.7–19.5) ng/ml after RT, *p* < 0.001 (Fig. 1). TGF-β1 and PDGF exhibited a strong correlation before RT (Spearman’s rho = 0.802) and after RT (rho = 0.817). The change in TGF-β1 also correlated with the change in PDGF (rho = 0.817) (Fig. 2). There was no significant correlation between change in TGF-β1 or PDGF and the time from surgery to RT (Additional file 1: Table S1) or radiation doses to the heart (Additional file 2: Table S2). Median time from surgery to start of RT was 56.0 (IQR 49.0–64.5) days.

### Transforming growth factor-beta 1, platelet-derived growth factor and cardiac function

#### TGF-β1 and PDGF levels and changes in TAPSE

Sixty-six of the 73 (90%) patients had echocardiography completed before and after RT. TAPSE declined by ≥15% in 20 patients and by < 15% in 46 patients. In the 20 patients with a ≥ 15% TAPSE decline, TAPSE was 25.0 (IQR 23.3–30.0) mm before RT and 20.5 (IQR 18.0–23.0) mm after RT, *p* < 0.001. However, in the 46 patients with a < 15% decline, the median TAPSE was stable with 22.5 (IQR 20.0–26.0) mm and 22.0 (IQR 19.0–25.3) mm (*p* = 0.298) before and after RT, respectively. The baseline TAPSE was significantly higher in the group with a ≥ 15% TAPSE decline than in the group with a < 15% decline, *p* = 0.021. The groups were similar in body mass index (BMI), age, smoking status, proportion of left-sided breast cancer, coronary artery disease, hypertension, and use of aromatase inhibitors (AI), tamoxifen, acetylsalicylic acid (ASA), statins, levothyroxine, diabetes medication, angiotensin converting enzyme (ACE) inhibitors or angiotensin reseptor blockers (ARB) and β-blockers (Additional file 3: Table S3).

**Fig. 3:**
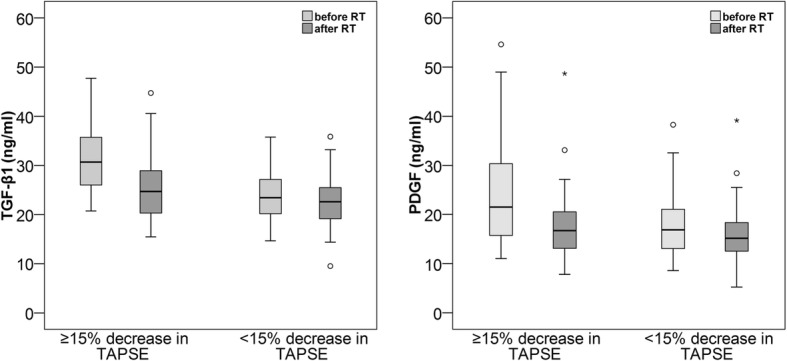
Baseline TGF-β1 and PDGF levels were higher, *p* < 0.001 and *p* = 0.020, respectively, and both decreased significantly, *p* < 0.001, in patients with a ≥ 15% decrease in TAPSE compared with patients with a < 15% decrease in TAPSE. TGF-β1 levels were stable, but PDGF levels decreased significantly, *p* = 0.005, in patients with a < 15% decrease in TAPSE

In the patients with a ≥ 15% decline in TAPSE, the median TGF-β1 level decreased from 30.7 (IQR 26.0–35.9) ng/ml before RT to 24.7 (IQR 20.0–29.8) ng/ml after RT, *p* < 0.001 (Fig. 3). TGF-β1 remained stable in patients with a < 15% decline in TAPSE, with a median TGF-β1 of 23.4 (IQR 20.1–27.3) ng/ml before RT and 22.6 (IQR 19.0–25.6) ng/ml after RT, *p* = 0.104. The baseline TGF-β1 level was also significantly higher, *p* < 0.001, in those with a ≥ 15% TAPSE decline than in those without. There was no correlation between change in TGF-β1 and the change in TAPSE (Additional file 4: Table S4). In a multivariable logistic regression analysis the change in TGF-β1 remained significant, OR 0.85 (95% CI 0.75–0.96) when age, hypertension and mean heart dose were included in the model to test variables associated with ≥15% and < 15% decline in TAPSE (Additional file 5: Table S5).

PDGF levels decreased significantly in both groups. In patients with a ≥ 15% decline in TAPSE, PDGF levels decreased from a median of 21.5 (IQR 15.7–31.2) ng/ml before RT to a median of 16.7 (IQR 12.9–20.9) ng/ml after RT, *p* < 0.001. In patients with a < 15% decline, PDGF levels decreased from a median of 16.9 (IQR 13.0–21.2) ng/ml before RT to a median of 15.1 (IQR 12.5–18.6) ng/ml after RT, *p* = 0.005 (Fig. 3). In addition, the baseline PDGF level was significantly higher, *p* = 0.020, in patients with a ≥ 15% decline than in those with a < 15% decline. The change in PDGF did not correlate with the change in TAPSE (Additional file 4: Table S4). In a multivariable logistic regression analysis the change in PDGF remained significant, OR 0.85 (95% CI 0.75–0.97) when age, hypertension and mean heart dose were included in the model (Additional file 6: Table S6).There was no difference in radiation doses to the heart between the groups with ≥15% or < 15% decline in TAPSE (Table 2).

**Table 2:** Radiation doses according to TAPSE decline

	≥15% decrease in TAPSE (*n* = 20)	< 15% decrease in TAPSE (*n* = 46)	
	Md (IQR)	Md (IQR)	*p*
Heart			
Dmean (Gy)	3.39 (0.8–4.2)	1.8 (0.8–3.5)	0.343
Dmax (Gy)	46.1 (5.3–49.0)	45.9 (11.8–47.5)	0.676
V45 (%)	0.2 (0–1.3)	0.1 (0–0.7)	0.630
V20 (%)	4.3 (0–5.2)	1.4 (0–4.8)	0.330
LAD			
Dmean (Gy)	23.7 (0.3–28.5)	10.3 (2.3–23.5)	0.414
Dmax (Gy)	44.1(0.7–48.1)	40.8 (5.0–46.1)	0.460
V45 (%)	0(0–5.2)	0(0–7.3)	0.663
V20 (%)	43.6 (0–67.6)	19.7(0–54.9)	0.193
Left ventricle			
Dmean (Gy)	4.6 (0.2–7.2)	2.7 (1.2–5.5)	0.273
Dmax (Gy)	44.6 (0.8–47.6)	44.1 (5.0–46.7)	0.691
V45 (%)	0.1 (0–2.0)	0 (0–0.4)	0.360
V20 (%)	6.0 (0–11.7)	1.7 (0–7.8)	0.192
V10 (%)	8.8 (0–15.1)	3.4 (0–11.0)	0.150
Right ventricle			
Dmean (Gy)	2.0 (0.8–7.2)	1.6 (0.9–2.9)	0.692
Dmax (Gy)	27.6 (3.1–43.4)	21.3 (3.3–42.8)	0.988
V45 (%)	0 (0–0)	0 (0–0)	0.925
V20 (%)	0.1 (0–1.3)	0 (0–1.3)	0.848
V10 (%)	0.7 (0–3.6)	0.1 (0–3.8)	0.714
Ipsilateral lung			
Dmean (Gy)	8.1 (6.4–9.1)	7.7 (6.2–9.0)	0.484
Dmax (Gy)	49.2 (46.2–52.7)	48.5 (47.1–51.6)	0.994

*Md* median*, IQR* interquartile range, *Dmean* mean radiation dose to the structure, *Dmax* maximum radiation dose within the structure, *V45* percentage of the structure volume receiving 45 Gy of radiation, *V20* percentage of the structure volume receiving 20 Gy of radiation, *V10* percentage of the structure volume receiving 10 Gy of radiation, *LAD* left anterior descending coronary artery

Fifty patients had left-sided breast cancer. TGF-β1 and PDGF behavior was similar in the left-sided patients as described above for the whole group. During RT, TGF-β1 levels decreased from 24.1 (IQR 20.9–29.8) ng/ml to 23.4 (IQR 19.4–26.9) ng/ml, *p* = 0.025, and PDGF levels decreased from 17.6 (IQR 13.4–22.8) ng/ml to 15.3 (IQR 12.7–19.8) ng/ml, *p* = 0.001. When the patients with left-sided breast cancer were grouped according to the ≥15% or < 15% decline in TAPSE, the mean radiation dose to the heart was higher in the group with a ≥ 15% decline than in those with a < 15% decline, with 3.9 (IQR 3.2–4.3) Gy and 2.2 (IQR 1.6–3.7) Gy received, respectively, *p* = 0.024. Similarly, the mean doses to the left descending coronary artery (LAD), 1.1 (IQR 0.4–1.5) Gy vs. 0.7 (IQR 0.4–0.9) Gy (*p* = 0.006), and the left ventricle, 7.0 (IQR 4.2–8.0) Gy vs. 3.8 (IQR 2.1–5.6) Gy (*p* = 0.005), were significantly higher in the ≥15% group compared to the < 15% group.

#### TGF-β1, PDGF and change in cIBS

Sixty-four of the 73 (88%) patients had cIBS measured by echocardiography. Twenty-nine patients had a ≥ 15% increase in cIBS, from a median of − 19.8 (IQR -22.6- -16.6) dB before RT to a median of − 13.3 (IQR -15.3- -9.5) dB after RT, *p* < 0.001. The group of 35 patients with a < 15% increase in cIBS had a significant decrease in cIBS from − 17.1 (IQR -21.5- -14.6) dB before RT to − 18.3 (IQR -24.0- -16.7) dB after RT, *p* = 0.033. The baseline cIBS values between the groups did not differ significantly, *p* = 0.257. Furthermore, the groups had similar baseline characteristics (Additional file 7: Table S7). There was a tendency for patients with a ≥ 15% increase to be older than those with a < 15% increase, 65 (IQR 59.5–69) years old and 62 (IQR 58–66) years old, respectively (*p* = 0.079). Additionally, smokers tended to more likely have a < 15% increase in cIBS (*p* = 0.063).

**Fig. 4:**
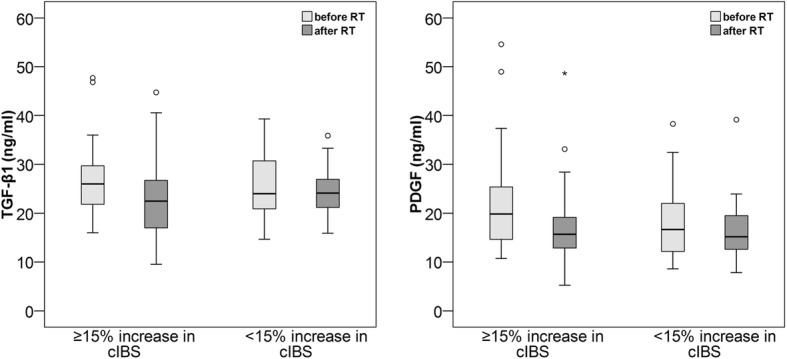
TGFβ-1 and PDGF levels decreased significantly in patients with a ≥ 15% increase in cIBS, *p* < 0.001 for both, but remain stable in patients with a < 15% increase in cIBS

In patients with a ≥ 15% increase in cIBS, the median TGF-β1 level decreased significantly from 26.0 (IQR 21.7–29.7) ng/ml before RT to 22.5 (16.6–26.7) ng/ml after RT (*p* < 0.001) (Fig. 4). TGF-β1 remained stable in patients with a < 15% increase in cIBS, with a median TGF-β1 level of 24.0 (IQR 20.7–31.4) ng/ml and 24.1 (IQR 21.1–22.5) ng/ml before and after RT, respectively (*p* = 0.149). The baseline TGF-β1 levels were similar in both groups, *p* = 0.518. There was no correlation between the change in TGF-β1and the change in cIBS (Additional file 4: Table S4). In a multivariable logistic regression analysis the change in TGF-β1 remained borderline significant, OR 0.91 (95% CI 0.82–1.00) when age, hypertension and mean heart dose were included in the model (Additional file 5: Table S5).

In addition to declining TGFβ-1 levels, a significant decrease in PDGF levels was observed from 19.8 (IQR 14.6–25.9) ng/ml before RT to 15.7 (IQR 12.8–20.2) ng/ml after RT, *p* < 0.001, in patients with a ≥ 15% increase in cIBS. There was no significant change in PDGF levels in patients with a < 15% increase in cIBS, with a median PDGF of 16.6 (IQR 11.7–22.6) before RT and 15.2 (IQR 12.6–19.8) after RT, *p* = 0.053. The baseline PDGF level tended to be higher in those with a ≥ 15% increase than in those with a < 15% increase in cIBS, *p* = 0.050. The change in PDGF did not correlate with the change in cIBS (Additional file 4: Table S4). In a multivariable logistic regression analysis change in PDGF remained significant, OR 0.88 (95% CI 0.78–0.99), when age, hypertension and mean heart dose were included in the model (Additional file 6: Table S6).

The radiation doses, especially those to the left side of the heart and to the ipsilateral lung, were higher in those with a ≥ 15% increase in cIBS than those with a < 15% increase in cIBS. Table 3 presents a detailed depiction of the radiation doses.

**Table 3:** Radiation doses according to the change in cIBS

Structure	≥15% increase in cIBS (*n* = 29)	< 15% increase in cIBS (*n* = 35)	
	Md (IQR)	Md (IQR)	*p*
Heart			
Dmean (Gy)	3.4 (1.1–4.2)	1.6 (0.8–2.7)	**0.011**
Dmax (Gy)	47.2 (15.0–48.8)	44.5 (6.4–47.1)	0.068
V45 (%)	0.5 (0–1.7)	0 (0–0.4)	**0.025**
V20 (%)	4.4 (0–6.2)	1.1 (0–2.4)	**0.005**
LAD			
Dmean (Gy)	22.9 (2.3–27.3)	7.3 (0.4–18.0)	**0.042**
Dmax (Gy)	45.9 (5.3–46.8)	36.3 (0.6–45.3)	**0.040**
V45 (%)	0.4 (0–13.9)	0 (0–0.3)	**0.008**
V20 (%)	42.7 (0–68.7)	8.9 (0–38.4)	**0.041**
Left ventricle			
Dmean (Gy)	4.9 (1.2–7.0)	2.3 (0.2–3.8)	**0.023**
Dmax (Gy)	45.8 (9.0–47.8)	41.7 (0.7–45.9)	0.080
V45 (%)	0.1 (0–2.9)	0 (0–0.2)	0.006
V20 (%)	6.7 (0–10.4)	1.3 (0–4.7)	**0.021**
V10 (%)	9.0 (0–15.0)	3.0 (0–6.5)	**0.006**
Right ventricle (*n* = 49)			
Dmean (Gy)	2.4 (1.1–3.1)	1.5 (0.9–2.3)	0.074
Dmax (Gy)	29.6 (3.6–44.0)	8.1 (3.0–39.3)	0.189
V45 (%)	0 (0–0)	0 (0–0)	0.150
V20 (%)	0.1 (0–2.7)	0 (0–0.4)	0.053
V10 (%)	0.9 (0–6.5)	0 (0–1.4)	**0.019**
Lung			
Dmean (Gy)	8.2 (7.6–9.6)	6.8 (5.5–8.2)	**0.001**
Dmax (Gy)	50.1 (47.9–56.7)	47.5 (46.4–49.7)	**0.009**

*Md* median*, IQR* interquartile range, *Dmean* mean radiation dose to the structure, *Dmax* maximum radiation dose within the structure, *V45* percentage of the structure volume receiving 45 Gy of radiation, *V20* percentage of the structure volume receiving 20 Gy of radiation, *V10* percentage of the structure volume receiving 10 Gy of radiation, *LAD* left anterior descending coronary artery

## Discussion

In this study, we demonstrated the behavior of serum TGF-β1 and PDGF during adjuvant RT for breast cancer. A small decline was observed in all patients, but the decline was most pronounced in patients with worsening cardiac function and structural changes observed in echocardiography. We also found a strong correlation between baseline TGF-β1 and PDGF levels and the change in TGF-β1 and PDGF levels during RT. This correlation is probably explained by the same origin of both cytokines, which are produced by macrophages, although TGF-β1 is additionally produced by endothelial and mesenchymal cells [1, 2]. This result suggests that the behavior of both cytokines depict the same phenomenon during adjuvant RT for breast cancer. The time from surgery to RT did not affect TGF-β1 and PDGF levels and change in these levels. This is probably because the wound was healed by the time RT started and the half-life of TGF-β1 and PDGF in serum is short [21].

### Transforming growth factor-beta 1 and cardiac function

It is generally accepted that TGF-β1 is a pro-fibrotic cytokine that initiates fibrosis in response to RT [1, 2]. Additionally, it plays a role in cardiac remodeling after myocardial infarction [3]. Earlier studies have shown that an increase in TGF-β1 during RT for non-small cell lung cancer is likely to be predictive for the development of radiation pneumonitis [6]. However, some studies do not confirm this finding, and in patients who do not develop radiation pneumonitis, a decrease in TGF-β1 levels is seen [22, 23]. In lung cancer, this decline is thought to represent a decrease in production of TGF-β1 by tumor cells. However, this explanation probably does not explain the decline in TGF-β1 in our study since our patients underwent breast conserving surgery or mastectomy and the likelihood of macroscopic tumor residual is extremely small.

The dynamics of TGF-β1 during adjuvant RT for breast cancer have not been previously reported; however, two studies found that an increased TGF-β1 level before RT was predictive of fibrosis of the breast [4, 5]. Neither of these studies reported the behavior of TGF-β1 during RT. In our patients with a 15% decline in TAPSE, the baseline TGF-β1 level was higher than that in patients without the decline, indicating an association between high TGF-β1 levels and right ventricular dysfunction induced by RT.

TGF-β1 also seems to be a marker of radiosensitivity. A decrease in TGF-β1 levels during RT is associated with a positive response to RT [24]. Additionally, in vitro experiments suggest that blockade of TGF-β1 during RT for non-small cell lung cancer and breast cancer increases radiosensitivity [25, 26]. Therefore, increased TGF-β1 levels seem to be a marker for both radioresistance and radiosensitivity, depending on the tissue in question. As the association of TGF-β1 and echocardiographic has not been studied during RT, we present a novel finding. Levels of TGF-β1 decreased significantly in patients with a decline in TAPSE and an increase in cIBS. TAPSE is in wide clinical use as a reliable measurement of the right ventricular function, and a decline in TAPSE correlates with poor cardiac prognosis in many patient groups [17, 20]. Myocardial reflectivity can be determined with off-line analysis of the echocardiography acquisition (cIBS). Even though the exact basis for the changes in cIBS are not completely understood, an increase in cIBS presents changes in three-dimensional myocardial structure due to factors such as tissue edema or interstitial fibrosis [27].

In studies of cardiac function after an experimental myocardial infarction in mice, blockade of TGF-β1 by an antibody increased mortality and left ventricular dilatation [28]. Another study concluded that early inhibition of TGFβ-1 was detrimental and that later inhibition was beneficial to the cardiac function of mice after an MI, which indicates that the role of TGFβ-1 may be different in various phases of the healing process [29]. In obese, hypertensive patients, an abundance of circulating TGF-β1 is associated with left ventricular filling abnormalities [30]. As concluded by a review conducted by Bujak, the role of TGF-β1 after an MI remains elusive [3].

### Platelet-derived growth factor and cardiac function

PDGF consists of two linked chains, designated A, B, C or D. It can be assembled as a hetero- or homodimer [8]. We measured the heterodimer PDGF-AB and found that RT induced a decrease in PDGF that was associated with a decrease in TAPSE and an increase in cIBS. The role of PDGF in long-term adverse effects of RT is not as extensively studied as is the role of TGFβ-1. During RT, PDGF levels were decreased in non-Hodgkin lymphoma patients with varying RT sites, some with preceding chemotherapy and some without [9]. In patients receiving chemotherapy and RT, PDGF and TGF-β1 levels remained unchanged [10]. Because the sites of RT varied, some patients had intact tumors and some patients had chemotherapy that also may influence cytokine levels, indicating that the studies were quite different from our study.

The effect of PDGF has also been studied through inhibition of the PDGF receptor. In mice, treatment with a PDGF receptor tyrosine kinase inhibitor, imatinib, attenuated the development of lung and skin fibrosis [11, 31]. The function of PDGF in cardiac tissue remains elusive, as blockade of PDGF receptor improved cardiac function [32], but injection of exogenous PDGF-AB or PDGF-BB improved heart function after an MI [33–35].

### Echocardiographic changes associate with radiation doses

In our earlier study, we reported changes in TAPSE during RT of left-sided breast cancer patients but found no association with radiation doses [19]. In this study, only patients with available serum samples were included. We found that patients with left-sided breast cancer that had a ≥ 15% decline in TAPSE had higher radiation doses to the whole heart, the left ventricle and the LAD than those with a < 15% decline in TAPSE. There were even more differences in the radiation doses to the whole heart or parts of the heart when patients were grouped according to ≥15% and < 15% increases in cIBS. An increase in cIBS represents an increase in the reflectivity of the cardiac tissue, probably due to structural changes caused by RT-induced inflammation. TAPSE is a parameter depicting longitudinal function of the right ventricle. Its decrease may portray inflammatory changes because the thinness of the right ventricle makes it more sensitive to RT-induced changes [19].

### Study limitations

The limitations to our study are that the population is rather small and the follow-up time is very short, as we only studied the changes that occurred during adjuvant RT. At this stage, we do not know if the echocardiographic changes are permanent or if they are associated with the development of fibrosis, which is thought to be responsible for the increased risk of cardiac morbidity after irradiation of the heart [36]. Thus, longer follow-up times are needed to determine whether the behavior of TGF-β1 and PDGF during adjuvant RT depict permanent damage to the heart.

## Conclusion

In this study, we demonstrated that RT induces a decrease in TGF-β1 and PDGF levels in accordance with worsening cardiac function and structural changes, namely, a decrease in TAPSE and an increase in cIBS. Additionally, higher baseline TGF-β1 and PDGF levels were associated with a decrease in TAPSE, possibly indicating a higher susceptibility to RT-induced cardiac changes. Decreases in TGF-β1 and PDGF levels and the association of these cytokines with echocardiographic changes could depict increased sensitivity of the heart to the effects of radiation. These novel findings are preliminary and need to be confirmed by more studies and longer follow-up, as serum biomarkers are an attractive, minimally invasive and easily available option to identify RT patients in need of closer cardiological follow-up.

## Additional files

Additional file 1: **Table S1.** Spearman’s correlation coefficients. RT radiotherapy, TFGβ transforming growth factor, PDGF platelet derived growth factor (DOCX 18 kb)

Additional file 2: **Table S2.** Spearman’s correlation coefficient between changes in TFG-β1 and PDGF and radiation doses. *Dmean,* mean radiation dose to the structure; *Dmax,* maximum radiation dose within the structure; *V45* percentage of the structure volume receiving 45 Gy of radiation; *V20*, percentage of the structure volume receiving 20 Gy of radiation; *V10*, percentage of the structure volume receiving 10 Gy of radiation; *LAD,* left anterior descending coronary artery. (DOCX 18 kb)

Additional file 3: **Table S3.** Baseline characteristic according to change in TAPSE. *TAPSE*, tricuspid annular plane systolic excursion; *BMI*, body mass index; *bc*, breast cancer; *Hypertension*, use of hypertension medication; *ASA*, low dose acetylsalicylic acid; *Diabetes*, use of diabetes medication; *ACE* angiotensin converting enzyme inhibitor; *ARB,* angiotensin II receptor blocker; *AI* aromatase inhibitor use. (DOCX 18 kb)

Additional file 4: **Table S4.** Spearman’s correlation coefficient between changes in TFG-β1, PDGF, TAPSE and cIBS. *TFG*-*β1*, transforming growth factor beta 1; *PDGF*, platelet derived growth factor; *TAPSE*, tricuspid annular plane systolic excursion; *cIBS*, pericardium calibrated integrated backscatter. (DOCX 17 kb)

Additional file 5: **Table S5.** Multivariable logistic regression analysis with change < 15% or ≥ 15% in TAPSE and cIBS. *TAPSE*, tricuspid annular plane systolic excursion; *cIBS*, pericardium calibrated integrated backscatter; *TFG*-*β1*, transforming growth factor beta 1 (DOCX 18 kb)

Additional file 6: **Table S6.** Multivariable logistic regression analysis with change < 15% or ≥ 15% in TAPSE and cIBS. *TAPSE*, tricuspid annular plane systolic excursion; *cIBS*, pericardium calibrated integrated backscatter; *PDGF,* platelet derived growth factor. (DOCX 18 kb)

Additional file 7: **Table S7.** Baseline characteristic according to change in cIBS. *cIBS*, pericardium calibrated integrated backscatter; *BMI*, body mass index; *bc*, breast cancer; *Hypertension*, use of hypertension medication; *ASA*, low dose acetylsalicylic acid; *Diabetes*, use of diabetes medication; *ACE* angiotensin converting enzyme inhibitor; *ARB,* angiotensin II receptor blocker; *AI* aromatase inhibitor use. (DOCX 18 kb)

## Abbreviations

AI: Aromatase inhibitor
ASA: Acetylsalicylic acid
BMI: Body mass index
cIBS: Pericardium calibrated integrated backscatter
DCIS: Ductal carcinoma in situ
IQR: Interquartile range
LAD: Left descending coronary artery
MI: Myocardial infarction
PDGF: Platelet derived growth factor
PTV: Planning target volume
RT: Radiotherapy
TAPSE: Tricuspid annular plane systolic excursion
TGF-β1: Transforming growth factor beta 1
