# Supplementary material for: Decreases in TGF-β1 and PDGF levels are associated with echocardiographic changes during adjuvant radiotherapy for breast cancer
Source: Radiat Oncol. 2018 Oct 19;13:201. doi: 10.1186/s13014-018-1150-7 (PMC6194684; doi:10.1186/s13014-018-1150-7)
Supplement: Supplementary file 1 — Table S1. Spearman’s correlation coefficients. RT radiotherapy, TFGβ transforming growth factor, PDGF platelet derived growth factor (DOCX 18 kb) [file 13014_2018_1150_MOESM1_ESM.docx]

**Additional file 1: Table S1** Spearman’s correlation coefficients

|  | Days from surgery to RT | TFG-β1 before RT | TFG-β1 after RT | change in TFG-β1 | PDGF before RT | PDGF after RT | change in PDGF |
| --- | --- | --- | --- | --- | --- | --- | --- |
| Days from surgery to RT | 1.000 |  |  |  |  |  |  |
| TFG-β1 before RT | 0.012 | 1.000 |  |  |  |  |  |
| TFG-β1 after RT | –0.264 | 0.400 | 1.000 |  |  |  |  |
| change in TFG-β1 | –0.159 | –0.592 | 0.409 | 1.000 |  |  |  |
| PDGF before RT | 0.026 | 0.802 | 0.304 | –0.453 | 1.000 |  |  |
| PDGF after RT | –0.160 | 0.378 | 0.758 | 0.278 | 0.589 | 1.000 |  |
| change in PDGF | –0.148 | –0.555 | 0.329 | 0.817 | –0.614 | 0.173 | 1.000 |

RT radiotherapy, TFGβ transforming growth factor, PDGF platelet derived growth factor
