# Supplementary material for: Decreases in TGF-β1 and PDGF levels are associated with echocardiographic changes during adjuvant radiotherapy for breast cancer
Source: Radiat Oncol. 2018 Oct 19;13:201. doi: 10.1186/s13014-018-1150-7 (PMC6194684; doi:10.1186/s13014-018-1150-7)
Supplement: Supplementary file 2 — Table S2. Spearman’s correlation coefficient between changes in TFG-β1 and PDGF and radiation doses. Dmean, mean radiation dose to the structure; Dmax, maximum radiation dose within the structure; V45 percentage of the structure volume receiving 45 Gy of radiation; V20, percentage of the structure volume receiving 20 Gy of radiation; V10, percentage of the structure volume receiving 10 Gy of radiation; LAD, left anterior descending coronary artery. (DOCX 18 kb) [file 13014_2018_1150_MOESM2_ESM.docx]

**Additional file 2: Table S2** Spearman’s correlation coefficient between changes in TFG-β1 and PDGF and radiation doses

|  | | change in TFGβ | change in PDGF |
| --- | --- | --- | --- |
| Heart | |  |  |
|  | Dmean (Gy) | –0.131 | –0.132 |
|  | Dmax (Gy) | –0.102 | –0.151 |
|  | V45 (%) | –0.171 | –0.213 |
|  | V20 (%) | –0.187 | –0.189 |
| LAD | |  |  |
|  | Dmean (Gy) | –0.135 | –0.119 |
|  | Dmax (Gy) | –0.125 | –0.154 |
|  | V45 (%) | –0.189 | –0.250 |
|  | V20 (%) | –0.154 | –0.126 |
| Left ventricle | |  |  |
|  | Dmean (Gy) | –0.129 | –0.133 |
|  | Dmax (Gy) | –0.058 | –0.104 |
|  | V45 (%) | –0.148 | –0.214 |
|  | V20 (%) | –0.147 | –0.139 |
|  | V10 (%) | –0.186 | –0.183 |
| Right ventricle | |  |  |
|  | Dmean (Gy) | –0.119 | –0.127 |
|  | Dmax (Gy) | –0.124 | –0.114 |
|  | V45 (%) | –0.205 | –0.249 |
|  | V20 (%) | –0.175 | –0.232 |
|  | V10 (%) | –0.152 | –0.170 |
| Ipsilateral lung | |  |  |
|  | Dmean (Gy) | –0.166 | –0.093 |
|  | Dmax (Gy) | –0.061 | –0.085 |

*Dmean,* mean radiation dose to the structure; *Dmax,* maximum radiation dose within the structure; *V45* percentage of the structure volume receiving 45 Gy of radiation; *V20*, percentage of the structure volume receiving 20 Gy of radiation; *V10*, percentage of the structure volume receiving 10 Gy of radiation; *LAD,* left anterior descending coronary artery
