# Supplementary material for: Decreases in TGF-β1 and PDGF levels are associated with echocardiographic changes during adjuvant radiotherapy for breast cancer
Source: Radiat Oncol. 2018 Oct 19;13:201. doi: 10.1186/s13014-018-1150-7 (PMC6194684; doi:10.1186/s13014-018-1150-7)
Supplement: Supplementary file 3 — Table S3. Baseline characteristic according to change in TAPSE. TAPSE, tricuspid annular plane systolic excursion; BMI, body mass index; bc, breast cancer; Hypertension, use of hypertension medication; ASA, low dose acetylsalicylic acid; Diabetes, use of diabetes medication; ACE angiotensin converting enzyme inhibitor; ARB, angiotensin II receptor blocker; AI aromatase inhibitor use. (DOCX 18 kb) [file 13014_2018_1150_MOESM3_ESM.docx]

**Additional file 3: Table S3** Baseline characteristic according to change in TAPSE

|  | ≥15% decrease in TAPSE | | <15% decrease in TAPSE | | p-value |
| --- | --- | --- | --- | --- | --- |
| BMI (IQR), kg/m² | 26.2 | (24.5-30.3) | 26.0 | (24.1-29.3) | 0.375 |
| Age (IQR) | 62 | (57-66) | 64 | (58-67) | 0.459 |
| Current smoker (%), n | 4 | (20.0) | 4 | (8.7) | 0.232 |
| Left-sided bc (%), n | 14 | (70.0) | 36 | (78.3) | 0.538 |
| CAD | 2 | (10.0) | 1 | (2.2) | 0.216 |
| Hypertension (%), n | 8 | (40.0) | 20 | (43.5) | 1.000 |
| ASA use (%), n | 2 | (10.0) | 5 | (10.9) | 1.000 |
| Statin use (%), n | 3 | (15.0) | 11 | (23.9) | 0.524 |
| Hypothyreosis (%), n | 3 | (15.0) | 8 | (17.4) | 1.000 |
| Diabetes (%), n | 2 | (10.0) | 4 | (9.5) | 1.000 |
| Beta-blocker use (%), n | 3 | (15.0.) | 8 | (17.4) | 1.000 |
| ACE or ARB use (%), n | 5 | (25.0) | 15 | (32.6) | 0.771 |
| AI (%), n | 7 | (35.0) | 17 | (37.0) | 1.000 |
| Tamoxifen (%), n | 2 | (10.0) | 3 | (6.5) | 0.635 |

*TAPSE*, tricuspid annular plane systolic excursion; *BMI*, body mass index; *bc*, breast cancer; *Hypertension*, use of hypertension medication; *ASA*, low dose acetylsalicylic acid; *Diabetes*, use of diabetes medication; *ACE* angiotensin converting enzyme inhibitor; *ARB,* angiotensin II receptor blocker; *AI* aromatase inhibitor use
