# Supplementary material for: Decreases in TGF-β1 and PDGF levels are associated with echocardiographic changes during adjuvant radiotherapy for breast cancer
Source: Radiat Oncol. 2018 Oct 19;13:201. doi: 10.1186/s13014-018-1150-7 (PMC6194684; doi:10.1186/s13014-018-1150-7)
Supplement: Supplementary file 4 — Table S4. Spearman’s correlation coefficient between changes in TFG-β1, PDGF, TAPSE and cIBS. TFG-β1, transforming growth factor beta 1; PDGF, platelet derived growth factor; TAPSE, tricuspid annular plane systolic excursion; cIBS, pericardium calibrated integrated backscatter. (DOCX 17 kb) [file 13014_2018_1150_MOESM4_ESM.docx]

**Additional file 4: Table S4** Spearman’s correlation coefficient between changes in TFG-β1, PDGF, TAPSE and cIBS

|  | change in TFG-β1 | change in PDGF | change in TAPSE (%) | change in cIBS (%) |
| --- | --- | --- | --- | --- |
| change in TFG-β1 | 1.000 |  |  |  |
| change in PDGF | 0.817 | 1.000 |  |  |
| change in TAPSE (%) | 0.157 | 0.106 | 1.000 |  |
| change in cIBS (%) | 0.294 | 0.270 | –0.096 | 1.000 |

*TFG*-*β1*, transforming growth factor beta 1; *PDGF*, platelet derived growth factor; *TAPSE*, tricuspid annular plane systolic excursion; *cIBS*, pericardium calibrated integrated backscatter
