# Supplementary material for: Decreases in TGF-β1 and PDGF levels are associated with echocardiographic changes during adjuvant radiotherapy for breast cancer
Source: Radiat Oncol. 2018 Oct 19;13:201. doi: 10.1186/s13014-018-1150-7 (PMC6194684; doi:10.1186/s13014-018-1150-7)
Supplement: Supplementary file 5 — Table S5. Multivariable logistic regression analysis with change < 15% or ≥ 15% in TAPSE and cIBS. TAPSE, tricuspid annular plane systolic excursion; cIBS, pericardium calibrated integrated backscatter; TFG-β1, transforming growth factor beta 1 (DOCX 18 kb) [file 13014_2018_1150_MOESM5_ESM.docx]

**Additional file 5: Table S5** Multivariable logistic regression analysis with change <15 % or ≥15 % in TAPSE and cIBS.

|  | Change in TAPSE <15 % or ≥15 % | | |  | Change in cIBS <15 % or ≥15 % | | |
| --- | --- | --- | --- | --- | --- | --- | --- |
|  | n | OR | (95% CI) |  | n | OR | (95% CI) |
| Age | 66 | 0.95 | (0.85-1.05) |  | 64 | 1.11 | (0.99-1.24) |
| Hypertension | 66 | 1.15 | (0.31-4.15) |  | 64 | 0.45 | (0.13-1.63) |
| Change in TFG-β1 | 66 | 0.85 | (0.75-0.96) |  | 64 | 0.91 | (0.82-1.00) |
| Mean heart dose | 66 | 1.23 | (0.87-1.73) |  | 64 | 1.57 | (1.07-2.29) |

*TAPSE*, tricuspid annular plane systolic excursion; *cIBS*, pericardium calibrated integrated backscatter; *TFG*-*β1*, transforming growth factor beta 1
