# Supplementary material for: Decreases in TGF-β1 and PDGF levels are associated with echocardiographic changes during adjuvant radiotherapy for breast cancer
Source: Radiat Oncol. 2018 Oct 19;13:201. doi: 10.1186/s13014-018-1150-7 (PMC6194684; doi:10.1186/s13014-018-1150-7)
Supplement: Supplementary file 6 — Table S6. Multivariable logistic regression analysis with change < 15% or ≥ 15% in TAPSE and cIBS. TAPSE, tricuspid annular plane systolic excursion; cIBS, pericardium calibrated integrated backscatter; PDGF, platelet derived growth factor. (DOCX 18 kb) [file 13014_2018_1150_MOESM6_ESM.docx]

**Additional file 6: Table S6.** Multivariable logistic regression analysis with change <15 % or ≥15 % in TAPSE and cIBS.

|  | Change in TAPSE ≥15 % or <15 % | | |  | Change in cIBS ≥15 % or <15 % | | |
| --- | --- | --- | --- | --- | --- | --- | --- |
|  | n | OR | (95% CI) |  | n | OR | (95% CI) |
| Age | 66 | 0.96 | (0.86-1.07) |  | 64 | 1.12 | (1.00-1.25) |
| Hypertension | 66 | 1.11 | (0.31-3.98) |  | 64 | 0.43 | (0.12-1.55) |
| change in PDGF | 66 | 0.85 | (0.75-0.97) |  | 64 | 0.88 | (0.78-0.99) |
| Mean heart dose | 66 | 1.20 | (0.85-1.69) |  | 64 | 1.53 | (1.04-2.24) |

*TAPSE*, tricuspid annular plane systolic excursion; *cIBS*, pericardium calibrated integrated backscatter; *PDGF,* platelet derived growth factor
