# Supplementary material for: Decreases in TGF-β1 and PDGF levels are associated with echocardiographic changes during adjuvant radiotherapy for breast cancer
Source: Radiat Oncol. 2018 Oct 19;13:201. doi: 10.1186/s13014-018-1150-7 (PMC6194684; doi:10.1186/s13014-018-1150-7)
Supplement: Supplementary file 7 — Table S7. Baseline characteristic according to change in cIBS. cIBS, pericardium calibrated integrated backscatter; BMI, body mass index; bc, breast cancer; Hypertension, use of hypertension medication; ASA, low dose acetylsalicylic acid; Diabetes, use of diabetes medication; ACE angiotensin converting enzyme inhibitor; ARB, angiotensin II receptor blocker; AI aromatase inhibitor use. (DOCX 18 kb) [file 13014_2018_1150_MOESM7_ESM.docx]

**Additional file 7: Table S7.** Baseline characteristic according to change in cIBS

|  | ≥15% increase in cIBS | | <15% increase in cIBS | | p-value |
| --- | --- | --- | --- | --- | --- |
| BMI (IQR), kg/m² | 26.8 | (24.3-30.2) | 25.7 | (24.2-28.7) | 0.419 |
| Age (IQR) | 65 | (60-69) | 62 | (58-66) | 0.079 |
| Current smoker (%), n | 1 | (3.4) | 7 | (20.0) | 0.063 |
| Left-sided bc (%), n | 23 | (79.3) | 24 | (68.6) | 0.402 |
| CAD (%), n | 1 | (3.4) | 2 | (5.7) | 1.000 |
| Hypertension (%), n | 11 | (37.9) | 16 | (45.7) | 0.615 |
| ASA (%), n | 3 | (10.3) | 3 | (8.6) | 1.000 |
| Statin use (%), n | 7 | (24.1) | 7 | (20.0) | 0.766 |
| Hypothyreosis (%), n | 4 | (13.8) | 7 | (20.0) | 0.741 |
| Diabetes (%), n | 2 | (7.4) | 3 | (9.1) | 1.000 |
| β-blockers (%), n | 6 | (20.7) | 5 | (14.3) | 0.526 |
| ACE or ARB use (%), n | 7 | (24.1) | 13 | (37.1) | 0.293 |
| AI (%), n | 9 | (31.0) | 15 | (42.9) | 0.438 |
| Tamoxifen (%), n | 4 | (13.8) | 2 | (5.7) | 0.397 |

*cIBS*, pericardium calibrated integrated backscatter; *BMI*, body mass index; *bc*, breast cancer; *Hypertension*, use of hypertension medication; *ASA*, low dose acetylsalicylic acid; *Diabetes*, use of diabetes medication; *ACE* angiotensin converting enzyme inhibitor; *ARB,* angiotensin II receptor blocker; *AI* aromatase inhibitor use
